# The Impact of the Inoculation of Phosphate-Solubilizing Bacteria *Pantoea agglomerans* on Phosphorus Availability and Bacterial Community Dynamics of a Semi-Arid Soil

**DOI:** 10.3390/microorganisms9081661

**Published:** 2021-08-04

**Authors:** Ilhem Saadouli, Amor Mosbah, Raoudha Ferjani, Panagiota Stathopoulou, Ioannis Galiatsatos, Elias Asimakis, Ramona Marasco, Daniele Daffonchio, George Tsiamis, Hadda-Imene Ouzari

**Affiliations:** 1 Laboratoire de Microorganismes et Biomolécules Actives (LR03ES03), Facultédes Sciences de Tunis, Université Tunis El Manar, 2092 Tunis, Tunisia; Ilhem.saadouli@hotmail.fr (I.S.); ferjaniraoudha@hotmail.fr (R.F.); 2 Higher Institute for Biotechnology (ISBST), LR Biotechnology and Bio-Geo Resources Valorization, University of Manouba, BVBGR-LR11ES31, Biotechpole Sidi Thabet, 2020 Ariana, Tunisia; amor.mosbah@isbst.uma.tn; 3 Laboratory of Systems Microbiology and Applied Genomics, Department of Environmental Engineering, University of Patras, 2 Seferi St., 30100 Agrinio, Greece; panstath@upatras.gr (P.S.); jgalia96@gmail.com (I.G.); eliasasim@gmail.com (E.A.); 4 Red Sea Research Center (RSRC), Biological and Environmental Sciences and Engineering Division (BESE), King Abdullah University of Science and Technology (KAUST), Thuwal 23955-6900, Saudi Arabia; ramona.marasco@kaust.edu.sa (R.M.); daniele.daffonchio@kaust.edu.sa (D.D.)

**Keywords:** available P, microbial inoculants, soil bacterial community composition, soil bacterial community structure, high-throughput sequencing

## Abstract

The bacterial genus *Pantoea* has been widely evaluated as promising bacteria to increase phosphorus (P) availability in soil. The aim of this study was to characterize the phosphate solubilizing (PS) activity of a *Pantoea agglomerans* strain and to evaluate the impact of its application in a semi-arid soil on phosphate availability and structure of the bacterial communities as a whole. An incubation experiment under close-to-natural soil environmental conditions was conducted for 15 days at 30 °C. High-throughput sequencing of the bacterial 16S rRNA gene was used to characterize and to compare the bacterial community structure of *P. agglomerans*-inoculated soil with non-inoculated control. Furthermore, a qPCR-based method was developed for detection and quantification of the functional genes related to the expression of mineral phosphate solubilization (MPS) phenotype in *P. agglomerans*. The results showed that in vitro solubilization of Ca_3_(PO_4_)_2_ by *P. agglomerans* strain was very efficient (980 mg/L), and it was associated with a drop in pH due to the secretion of gluconic acid; these changes were concomitant with the detection of *gdh* and *pqqC* genes. Moreover, *P. agglomerans* inoculum application significantly increased the content of available P in semi-arid soil by 69%. Metagenomic analyses showed that *P. agglomerans* treatment modified the overall edaphic bacterial community, significantly impacting its structure and composition. In particular, during *P. agglomerans* inoculation the relative abundance of bacteria belonging to Firmicutes (mainly *Bacilli* class) significantly increased, whereas the abundance of Actinobacteria together with Acidobacteria and Chloroflexi phyla decreased. Furthermore, genera known for their phosphate solubilizing activity, such as *Aneurinibacillus*, *Lysinibacillus*, *Enterococcus*, and *Pontibacter,* were exclusively detected in *P. agglomerans*-treated soil. Pearson’s correlation analysis revealed that changes in soil bacterial community composition were closely affected by soil characteristics, such as pH and available P. This study explores the effect of the inoculation of *P. agglomerans* on the bacterial community structure of a semi-arid soil. The effectiveness in improving the phosphate availability and modification in soil bacterial community suggested that *P. agglomerans* represent a promising environmental-friendly biofertilizer in arid and semi-arid ecosystems.

## 1. Introduction

Phosphorus (P) makes up 0.7% of the Earth’s lithosphere and represents the eleventh most abundant element [1]. However, P is the most important key element next to nitrogen that often limits plant productivity [2]. Indeed, only 0.1% of the total P exists in a soluble form available for plant uptake [3] because of its complexation with cations like iron, aluminum, and calcium which can be regarded as an unavailable form for plants uptake.

Chemical phosphate fertilizer has been used as a source of replenishing soil P. The main source of manufacturing phosphate fertilizers is mined rock phosphate, which in turn is non-renewable and may only last for 100–400 years [4,5]. In addition to resource issues, chemical phosphate fertilizers represent the main cost of agricultural production and can cause environmental complications, including reduction of crop yield, water quality, and waterway eutrophication [4], along with changes in the edaphic microbial communities [6]. In addition, the repeated and injudicious applications of chemical P fertilizers may also lead to soil quality deterioration, related to soil acidification and soil structural degradation [7].

Due to the detrimental effects that are associated with chemical P fertilizers, there have been substantial efforts towards the search for environmentally compatible and economically feasible alternative strategies for improving crop production in low or P-deficient soils [8]. The use of phosphate-solubilizing bacteria (PSB)-based bio-fertilizers in agricultural soils is considered an environment-friendly alternative to replace agrochemical-based P fertilizers [2].

The use of PSB has been widely studied in recent years. In this way, many bacteria classified as PSB strains are used as biofertilizer inoculants in agriculture to improve crop P nutrition and increase crop yield, although with varied and inconsistent efficacy. PSB are emerging as key factors for P dynamics in soils and offer cost-effective and sustainable approach for smallholder farmers to overcome P limitations in soil [2].

The mineral phosphate solubilization (MPS) mechanism mediated by bacteria—mainly Gram-negative species—implies the secretion of organic acids through direct oxidation of glucose (DOPG; non-phosphorylating oxidation) into gluconic acid; gluconate is further oxidized to 2-ketogluconic acid by gluconate dehydrogenase (GADH) [9]. Gluconate is considered the key driver of phosphate solubilization (PS). The activity of glucose dehydrogenase (*gdh*) requires pyrroloquinoline quinone (PQQ) as cofactor, whose biosynthesis involves a *pqq* operon consisting of at least six to seven genes [10,11]. Although *pqq* genes are highly conserved in several bacterial species [12], the role of the protein *PqqC* (pyrroloquinoline quinone synthase C) is the only one to have been dilucidated; meanwhile, proposed functions have been assigned for most of the other genes [13]. The *pqqC* gene catalyzes the final step of the PQQ biosynthesis, namely, cyclization and oxidation of the intermediate 3a-(2-amino-2-carboxy-ethyl)-4,5-dioxo-4,5,6,7,8,9-hexahydroquinoline-7,9-dicarboxylic acid to PQQ [14].

Many different bacteria carry genes involved in the MPS mechanism, but little is known about their ecology and activity in soils. An important group of bacteria belonging to the *Enterobacteriaceae* family showed high P solubilization activity [15]. Among these, members of the genus *Pantoea* have been extensively studied [15]. *Pantoea* comprises several species that have showed prominent environmental versatility and adaptability, and possess a variety of biodegradative capabilities [16,17,18,19,20]. Numerous studies demonstrated that *Pantoea* possess genes involved in the expression of the MPS phenotype [20,21,22]. The majority of these studies are usually based on artificial media amended with a source of inorganic phosphate, such as Ca_3_(PO_4_)_2_ [17]. Compared to artificial media, the physiological state and metabolism of a bacterium grown in natural soil could be different, influencing the effectiveness of the PS observed in vitro. Moreover, PSB inoculated in natural soil must interact and compete with the indigenous edaphic microbial community, possibly impacting its structure and composition. To the best of our knowledge, there has been no report to date on the effects of *Pantoea* addition on the soil microbial community. Here, we investigate the effects of PSB application in natural arid soil on: (1) the availability of P and (2) the diversity and composition of the indigenous edaphic bacterial community. We isolated our PSB strains (*P. agglomerans* V8R67) from the rhizosphere of date palm (*Phoenix dactylifera* L.) cultivated in the arid oasis of Ksar Ghilane (Tunisia). We characterized the MPS pathway by the detection of *gdh* and *pqqC* genes and by evaluating its activity in vitro. Microcosm experiments were further conducted using agricultural soil with low available P, inoculated and non-inoculated with *P. agglomerans.* The structure and composition of the bacterial community exposed to *P. agglomerans* were evaluated by high-throughput amplicon sequencing of the bacterial 16S rRNA gene; prevalence and expression of genes *gdh* and *pqqC* were also evaluated by qPCR. This study will provide useful information regarding the effect of biofertilizer inocula on the soil bacterial communities and possibly on the multifunctionality of the soil ecosystem.

## 2. Materials and Methods

### 2.1. Phosphate-Solubilizing Strain Origin and Phylogenetic Identification

The strain used in this study (*Pantoea agglomerans* V8R67) was originally isolated from the rhizospheric soil of date palm (*Phoenix dactylifera* L.) in an oasis in southern Tunisia (Ksar Ghilane; N 32°58′56.040″ E 09°38′11.287″, alt. 6.0 m) and selected according to its ability to solubilize P in vitro [23]. The gene-encoding bacterial 16S rRNA was amplified from the strain by colony PCR using the bacterial universal primers 27F 5′-AGRGTTTGATCMTGGCTCAG-3′ and 1492R 5′-GGTTACCTTGTTACGACTT-3′ [24]. PCR reactions were carried out in 20 μL volume of reaction mixture, which included final concentrations of 1× reaction buffer (Kapa), 1 mM MgCl_2_, 0.5 mM dNTPs, 0.5 mM of each primer, 0.5 U/μL of Taq (Kapa), and 1 μL of template DNA (~100 ng). A PCR reaction was performed by an initial step at 95 °C for 5 min in order to denature DNA, followed by 35 cycles of 30 s denaturation at 94 °C, 30 s primer annealing at 55 °C, and 90 s DNA chain extension at 72 °C. The PCR was completed by a final extension at 72 °C for 10 min. The PCR products were precipitated with polyethylene glycol (PEG) [25]. Both strands were sequenced using the Big Dye Terminator v3.1 Cycle Sequencing Kit (Life Technologies Corporation, Austin, TX, USA) in a 3500 Genetic Analyzer (Life Technologies, Singapore). Sequence similarities were found by BLAST analysis [26] using the GenBank DNA database.

### 2.2. Characterization of the In Vitro Mineral Phosphate Solubilization (MPS) Activity in P. agglomerans V8R67

For the qualitative estimation of inorganic P solubilization, the strain was spot inoculated on National Botanical Research Institute’s Phosphate (NBRIP) [27] agar medium, containing insoluble tricalcium phosphate Ca_3_(PO_4_)_2_ for 3 days. The P solubilization is indicated by a clear zone around the colony. For quantitative estimation of inorganic P solubilization, the strain was inoculated in 20 mL vials containing NBRIP media and incubated at 30 °C for 6 days with shaking (250 rpm). Cell growth rate and medium acidification were monitored during the incubation; un-inoculated NBRIP medium was used as negative control. The broths were centrifuged at 13,000 rpm for 10 min to remove suspended particles of insoluble Ca_3_(PO_4_)_2_ and obtain a clear supernatant. Triplicate aliquots of the supernatant (100 μL) were transferred into clean, dry, acid-washed test tubes. Soluble phosphate in the culture broth was determined by using the vanadomolybdo phosphoric acid colorimetric method [28]. Qualitative detection of organic acids was performed by thin-layer chromatography (TLC) with the procedure described by Pérez et al. [29].

### 2.3. Detection of Genes Involved in the Expression of the MPS Phenotype in P. agglomerans V8R67

The presence of genes involved in the MPS phenotype was examined by using PCR in *P. agglomerans* V8R67. One of the most important mechanisms of phosphate solubilization by *Pantoea* spp. is the biosynthesis/secretion of gluconic acid that requires the enzyme glucose dehydrogenase (*gdh*) and its cofactor pyrroloquinolinequinone (PQQ; biosynthetic gene, *pqqC*) [22]. The *gdh* and *pqqC* genes were amplified using the primers listed in Supplementary Table S1, starting from the DNA previously extracted from the strain. PCR reactions were carried out in 20 μL volume of reaction mixture, which included final concentrations of 1× reaction buffer (Kapa), 1 mM MgCl_2_, 0.5 mM dNTPs, 0.5 mM of each primer, 0.5 U/μL of Taq (Kapa) and 1 μL of template DNA. The temperature profile for PCR-*gdh* and *pqqC* was: an initial cycle at 95 °C for 5 min, followed by 35 cycles at 94 °C for 30 s, at 57 °C for 30 s, at 72 °C for 2 min, and a final step of 72 °C for 10 min. Sequence similarities were found by BLAST analysis [26] using the GenBank DNA database (http://www.ncbi.nih.gov, accessed on 15 September 2019). Phylogenetic analysis of the *gdh* and *pqqC* gene sequences were conducted with Molecular Evolutionary Genetics Analysis (MEGA) software, version 5 [30]. Trees were constructed using neighbor-joining method [31].

### 2.4. Collection of Soil for Microcosm Test

Soil used for the preparation of microcosms was collected in an agricultural field situated in Sidi Bouzid, region located in the west center of Tunisia (34.5775° N, 9.8419° E), recently devoted to agriculture [32]. The soil was classified as semi-arid with low available phosphate compared to other soil in Tunisia [33]. Soil samples were collected from the top 20 cm of the soil profile following the procedures described by the International Standardization Organization (ISO) for collection and handling of soil samples (ISO 10381-6, 2009). After sampling, soil was mixed thoroughly for the subsequent incubation experiment, air-dried at room temperature, sieved (<2 mm), and stored at 4 °C for 1 week before starting the experiment. An aliquot of the collected samples was dried at room temperature and subjected to the physicochemical analyses as previously described by Moustarhfer et al. [34]. The properties of the soil are listed in Supplementary Table S2.

### 2.5. Soil Microcosm Setup

Soil microcosms were constructed in triplicate to evaluate the effects of *P. agglomerans* V8R67 addition to soil on P availability and edaphic bacterial community. A total of twelve pots were filled with 30 g of dry soil. Six of them were inoculated with 5 mL of *P. agglomerans* suspension of bacterial cells prepared in sterile saline solution (0.85% NaCl) at turbidity of 0.5 McFarland (~1.5 ×10^8^ cells/mL); the remaining pots were inoculated only with saline solution and used as controls. In addition, the same experimental design was applied to another twelve pots with capacity of 100 mL, prepared with sterile soil in order to prove that the increase in available phosphate level is due to the activity of *P. agglomerans*. Sterilization was performed by autoclaving the soil at 121 °C for 30 min for three cycles. One gram of soil was taken from 1 cm depth from each pot and the serial dilution was applied plated on TSA medium to check the efficacy of sterilization. All the microcosms were incubated for 15 days in an incubator with a 15/9 h day/night cycle at 30 °C temperature and 55% of relative humidity.

Samples of soils were collected from all the pots at the day of application (time 0, T0) and 15 days later (time 15, T15) by using sterile tools, and stored at −80 °C until processed for DNA and RNA extraction. The remaining soil was air-dried to determine the pH and available P content. The pH (soil/water ratio 1:2.5) was determined using a pH meter [35]. Available P was extracted with a 0.5 M NaHCO_3_ solution, adjusted to pH 8.5 [36].

### 2.6. Quantification of Genes Involved in the Expression of the MPS Phenotype in P. agglomerans V8R67 in Soil Microcosms

RNA was extracted from 0.5 g of soil by using Soil Total RNA Purification Kit (Norgen) according to the manufacturer’s instructions and cDNA was synthesized by reverse transcription using a PrimeScript first-strand cDNA synthesis kit (Takara). The absence of DNA contamination in RNA extracts was tested by PCR before reverse transcription amplification. We performed qPCR on the obtained cDNA to evaluate the expression and quantification of *P. agglomerans gdh* and *pqqC* genes. The designed primers used for the qPCR experiments are presented in Supplementary Table S1. Reactions were performed using the KAPA SYBR FAST qPCR Kit (KAPA Biosystems, UK) in 10 μL (2 replicates for each reaction) containing: 5 μL 2× KAPA SYBR FAST qPCR Master Mix Universal, 0.08 μL from each primer (25 μM), 4.34 μL water, and 0.5 μL total cDNA (~100 ng). qPCR runs were conducted in MJ Research Opticon 2 (MJ Research, Waltham, MA, USA) under the following conditions: one cycle at 95 °C for 5 min as enzyme activation, followed by 35 cycles of denaturation at 95 °C for 30 s, annealing at 57 °C for 30 s, and extension at 72 °C for 30 s. The reaction specificity was determined for each reaction by using a melting-curve analysis of the PCR product. Amplifications were carried out in a 96-well plate and each biological sample had a minimum of three replicates. Statistical significance was determined using the t-test. Internal standard curves were generated for each primer set by cloning the amplicon of each gene into a pGEM-T Easy Vector (Promega, Madison, USA) according to the manufacturer’s instructions. All assays were carried out in duplicate, and replicates were averaged for each sample. Negative controls were included in all amplification reactions.

### 2.7. Total Bacterial DNA Extraction, PCR Amplification and Purification

Total soil DNA was extracted from 0.25 g of microcosm soil samples using the Power Soil DNA Isolation Kit (MOBIO Laboratories Inc., Carlsbad, CA, USA) according to the manufacturer’s instructions. Total DNA concentration and quality (A260/A280) were estimated using a Quawell Q5000 micro-volume UV-Vis spectrophotometer. The purified DNA was stored at −20 °C for subsequent amplification by polymerase chain reaction (PCR) and Illumina MiSeq sequencing. A fragment of approximately 460 bp belonging to the V3-V4 region of the bacterial 16S rRNA gene was amplified by PCR using the universal primer set U341FMiSeq 5′-CCTACGGGRSGCAGCAG-3′ and 805RMiSeq 5′-GACTACHVGGGTATCTAATC C-3′ [36]. Amplification was performed using KAPA HiFiHot-Start PCR Kit (Kapa Biosystems). Each 25 μL reaction contained 5 μL of KAPA HiFi Fidelity Buffer (5×), 0.7 μL of dNTPs solution (10 mM each), 0.7 μL of each primer solution (10 μM), 0.3 μL of KAPA HiFi Hot-Start DNA Polymerase solution (1 U/μL), 1 μL from the template DNA solution and 16.6 μL of sterile deionized water. The PCR protocol included an initial denaturation step at 95 °C for 3 min, followed by 30 cycles of denaturation at 98 °C for 20 s, annealing at 60 °C for 15 s, and extension at 72 °C for 45 s. The reaction was terminated with a final extension step at 72 °C for 1 min. For each set of PCR reactions performed, the appropriate negative and positive controls were also prepared. The approximately 550 bp amplification products (size increase due to the incorporation of the 50-mer Illumina primers) were electrophoresed on a 1.5% *w*/*v* agarose gel and visualized in Bio-Rad’s Gel Doc™ XR+ system. Positive PCR products were purified with a 20% PEG, 2.5 M NaCl solution, centrifuged at 14,000× *g* for 20 min and the precipitate was washed twice with 125 μL of a 70% *v*/*v* ethanol solution and centrifuged at 14,000× *g* for 10 min as previously described [37]. The dried precipitates were suspended in 15 μL of sterile deionized water and the concentration was measured with a Quawell Q5000 micro-volume UV-Vis spectrophotometer.

### 2.8. Indexing PCR and Purification

The purified PCR products were diluted to a final concentration of 10 ng/μL and submitted to indexing PCR in order to incorporate the Illumina adapters (barcodes). During indexing PCR, each sample was amplified with a unique combination of index primers. Amplification was performed in 50 μL reactions using the KAPA HiFiHot-Start PCR Kit. Each reaction contained 10 μL of KAPA HiFi Fidelity Buffer (5×), 1.5 μL of dNTPs solution (10 mM each), 5 μL of the forward index primer (10 μM), 5 μL of the reverse index primer (10 μΜ), 1 μL of KAPA HiFiHot-Start DNA Polymerase (1 U/μL), 2 μL from the diluted PCR product (10 ng/μL), and 25.5 μL of sterile deionized water. The PCR program comprised an initial denaturation step at 95 °C for 3 min, followed by 8 cycles of denaturation at 95 °C for 30 s, annealing at 55 °C for 30 s, and extension at 72 °C for 30 s. The reaction was terminated with a final extension step at 72 °C for 5 min. The resulting amplicons were purified using Macherey-Nagel’s NucleoMag^®^ NGS Clean-up and Size Selection kit (MACHEREY-NAGEL GmbH & Co, Düren, Germany) according to the manufacturer’s recommendations. Purified samples were suspended in 30 μL of sterile deionized water and their concentration was measured with a Quawell Q5000 micro-volume UV-Vis spectrophotometer (Quawell, San Jose, CA, USA). All samples were diluted to a final concentration of 8 nM and mixed equimolarly.

### 2.9. Illumina Sequencing and Data Analysis

The library was sequenced on an Illumina MiSeq sequencing platform by Macrogen (Korea). Sequencing reads were de-multiplexed and converted to FASTQ. The Illumina adapters were removed using Illumina standard algorithms. Paired-end reads were assembled, trimmed by length, and further corrected using the usearch -fastq_mergepairs option. Analysis of reads was performed using usearch v.10 [38]. The quality of the assembled sequences was further improved using the -fastq_filter, followed by finding unique read sequences and abundances by using the -fastx_uniques option. Sequences were clustered into operational taxonomic units (OTUs) using the -cluster_otus command [39]. Chimeras were removed using the -unoise3 option [40]. Taxonomy was assigned using the SILVA 16S rRNA gene database (release 119) [41].

Alpha-diversity indices were calculated based on the rarefied OTUs table at a depth of 11,910 sequences/sample (Supplementary Figure S1). Species richness was estimated with Chao1 [42] and ACE indices [43], whereas species diversity was calculated with the use of Shannon’s and Simpson’s reciprocal (1/D) indices. Alpha-diversity comparisons were performed using analyses of variances (ANOVAs) in a factorial design followed by the Tukey HSD test (*p* < 0.05). Between-sample (Beta-diversity) was calculated using Bray–Curtis similarity [44] on square root transformed data, and principal coordinates analysis (PCoA) [45] was performed on the resulting distance matrix. Shared and exclusive OTUs (and their relative distribution) across treatments (soil inoculated with *P. agglomerans* and related control) were calculated for each sampling time (time 0 and 15) in R using the package Venn Diagram [46]; differential abundance of OTUs (2 fold-change) was also evaluated to determine OTUs enriched/depleted across treatments at the two sampling times by using package DEseq2 in R [47]. In addition, to identify indicator features in *P. agglomerans*-treated and control sample soil, the linear discriminant analysis (LDA) effect size (LEfSe) method was used [48]. The LDA was performed using a one-against-all strategy, an alpha significance level of 0.05, and an effect-size threshold of 4 for all distinctive taxa.

We performed a distance-based linear model permutation test to evaluate the influence of soil environmental factors (soil pH and available P) on the bacterial community structure. The Bray–Curtis distance matrix was used as the resemblance measure in DistLM procedures. The R^2^ was used as a selection criterion to permit the fitting of the explanatory environmental variables in the model. Results were visualized with a distance-based redundancy analysis (dbRDA). The analysis was performed using the PERMANOVA+ plugin utilized through PRIMER 6 [49,50]. In addition, the Minitab software was also used to conduct the Pearson correlation analysis to identify correlations between environmental factors and the relative abundances of abundant phyla results.

## 3. Results

### 3.1. Identification of PSB Pantoea Agglomerans V8R67 and Characterization of Its MPS Ability

**Figure 1:**
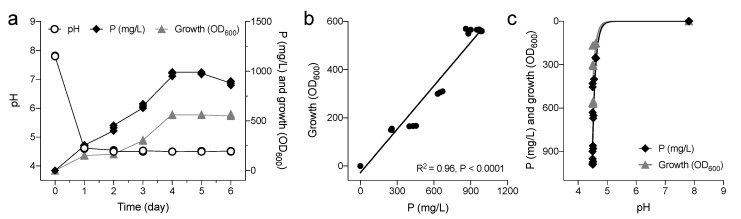
(**a**) Growth of *P. agglomerans* in NBRIP medium containing 5 g/L tri-calcium phosphate [Ca_3_(PO_4_)_2_]. Changes in soluble P, bacterial cell growth, and medium pH are reported; values of three independent readings are showed. (**b**) Relationship between soluble-P and bacterial growth; Pearson correlation *p*-values and R^2^ are reported in the graph. (**c**) Relationship between modification in pH and bacterial growth/soluble-P.

Genotypic identification of the selected strain was performed by analysis of its nucleotide sequences corresponding to the bacterial 16S rRNA gene. The strain was closely related to *P. agglomerans*. The DNA sequence of the 16S rRNA gene of this strain showed 99.86% and 99.79% identity to the ones of *P. agglomerans* strain S20_PA1R (GenBank accession no. MK883101.1) and of *P. brenneri* strain IHBB 9376 (GenBank accession no. KU921568.1), respectively. Cells of *P. agglomerans* V8R67 inoculated in NBRIP medium containing Ca_3_(PO_4_)_2_ as the sole P source started to grow exponentially after an initial log phase of about 3 days and reached the stationary phase after 4 days of growth (Figure 1a). PS increased with the increase of culture time, reaching a maximum of 990 mg/L at 4 days of growth (Figure 1a); bacterial growth and PS were positively correlated (Pearson correlation: R^2^ = 0.96, *p* < 0.0001; Figure 1b). The culture pH decreased from 7.8 to 4.65 during the period of the linear increase of PS (Figure 1a); such changes were related to both bacterial growth and PS activity (R^2^ = 0.6188 and R^2^ = 0.7595, respectively; Figure 1c), suggesting the involvement of organic acids secretion by *P. agglomerans*. This hypothesis was confirmed by TLC analysis. Spot with pure gluconic acid was detected in our culture supernatant, revealing that the solubilization of P mediated by *P. agglomerans* was caused by the secretion of organic acids in the culture medium.

The ability of *P. agglomerans* V8R67 to produce gluconic acid was further evaluated by amplifying the *gdh* and *pqqC* genes (Supplementary Figure S2). Amplification results showed the presence of bands with the expected size for both the *pqqC* (600 bp) and *gdh* (600 bp) genes. Partial sequencing of these PCR products confirmed that the two bands obtained from V8R67 correspond to the *pqqC* and *gdh* homologs from *P. agglomerans* strain C410P1 (CP016889) and *P. agglomerans* strain L15 (CP034148), respectively (Supplementary Figure S3).

### 3.2. Effects of P. agglomerans V8R67 Inoculum Application on the Availability of P and Diversity of Edaphic Bacterial Community

**Figure 2:**
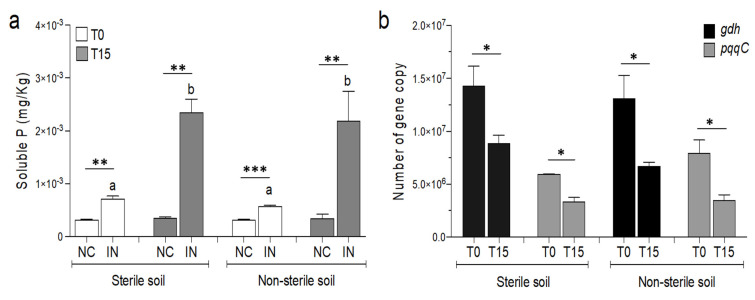
(**a**) Soluble P in *P. agglomerans* inoculated sterile and non-sterile soil at 0 and 15 days. NC: uninoculated soil; IN: inoculated soil; 0: day of the inoculum; 15: 15 days after inoculation; *t*-student test was performed to compare NC and IN at each time and different soils; statistically significant differences are indicated with the asterisks (**, *p* < 0.01; ***, *p* < 0.001). ANOVA multi-comparison was also performed to evaluate changes in NC and IN due to the treatment; different letters indicate significant differences (*p* < 0.01). (**b**) Quantification of the *gdh* and *pqqC* genes in sterile and non-sterile soils inoculated with *P. agglomerans* at 0 and 15 days; *t*-student test was performed to compare number of genes at 0 (T0) and 15 (T15) days for each gene (*, *p* < 0.05).

The addition of *P. agglomerans* V8R67 strain in the soil significantly (*t*-test student, *p* < 0.05) increased available P in the soil at the beginning and after 15 days of incubation (Figure 2a). This effect was observed in both sterile and non-sterile soils, with increase in the available P reaching up to 69% and 73% compared to the respective controls at the end of the incubation (Figure 2a). Notably, no significant differences were observed among sterile and non-sterile soils treated with the strain at each time (0 and 15 days). Despite the increase in soluble P in treated soils, the copy numbers of *gdh* and *pqqC* genes of active *P. agglomerans* tended to decrease during the incubation time (15-days) in both sterile and non-sterile soils, indicating a decline of the activity and cell quantity of the introduced bacteria over time (Figure 2b).

**Figure 3:**
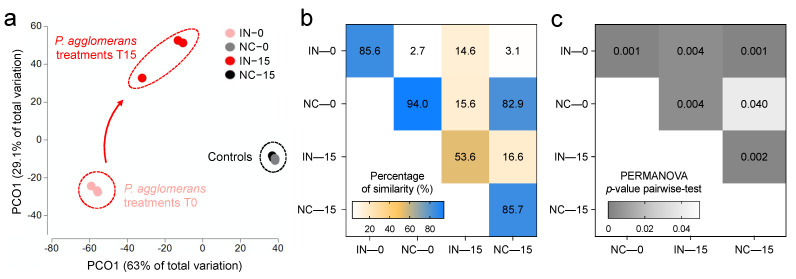
(**a**) Principal coordinate analysis (PCoA) of soil bacterial community structure of control and *P. agglomerans*-inoculated soil collected at 0 days and 15 days. Variance explained by each PCοA axis is given in parentheses. (**b**,**c**) Heatmaps showing the average values of similarity (%) and PERMANOVA *p*-values of pairwise comparisons across samples categories, respectively. NC: control; IN: inoculated; 0: 0 day; 15: 15 days.

The PCoA analysis indicated that the first two principal coordinates accounted for 63% and 29.1% of the variation within the Bray–Curtis similarity matrix, respectively (Figure 3a). The three replicates of each treatment were consistently located close to each other in the ordination space of PCoA, indicating reduced dispersion. Notably, the bacterial communities subjected to *P. agglomerans* treatment were separated from those of control samples (Figure 3a), showing an average similarity of 2.7% and 16.6% at 0 and 15 days, respectively (Figure 3b). In both controls and the *P. agglomerans* treatments, the incubation time was a significant differentiation factor of the bacterial communities (*p* = 0.04 and *p* = 0.004, respectively; Figure 3c); such separation was more evident and pronounced in the inoculated soils.

The analysis of alpha-diversity revealed that the inoculation with our strain (time 0) drastically reduced the diversity of bacterial communities in terms of number of OTUs (richness), and their diversity and distribution (Simpson and Shannon; Table 1; Supplementary Figure S4). This effect was mitigated during the incubation time, and at 15 days, the inoculated soil samples exhibited similar species richness and diversity indices compared to the control group (Table 1). However, as showed by the beta-diversity analysis, the inoculum determined the differentiation of soil bacterial communities that present up to 45% of specific-OTUs and have 55% of OTUs in common with the control (Supplementary Figure S4).

### 3.3. P. agglomerans Treatment Influences the Relative Abundance of Specific Taxa

**Figure 4:**
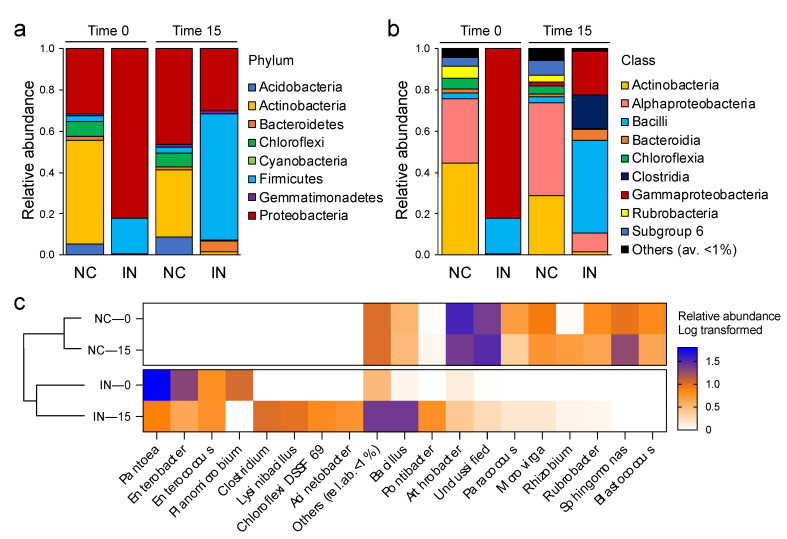
Percentage of relative abundance of bacterial (**a**) phyla, (**b**) classes, and (**c**) genera in control (NC) and *P. agglomerans* inoculated (IN) soils samples at 0 and 15 days. Relative abundance of genera is reported as log-transformed. For each treatment values are expressed as the average of the three replicates.

The analysis of the bacterial community revealed the presence of seven phyla (Figure 4a, Supplementary Table S3): Proteobacteria, Firmicutes, Actinobacteria, Acidobacteria, Chloroflexi, Bacteroidetes, and Gemmatimonadetes. *P. agglomerans* application promoted the presence of Firmicutes and Bacteroidetes which increased from 2.9% to 61.99% and from 1.64% to 5.19%, respectively, compared to the control (*p* < 0.05). By contrast, the phyla Chloroflexi and Actinobacteria significantly decreased in *P. agglomerans*-treated soil compared to non-treated soils, from 6.73% to 0.45% and from 32.26% to 1.65%, respectively (*p* < 0.05). Additionally, the treatment induced the loss of Acidobacteria, while the Gemmatimonadetes phylum was not affected. We also evaluated the treatment effect at the class and genus levels (Figure 4b,c and Supplementary Tables S3 and S4). While the relative abundance of *Alphaproteobacteria*, *Rubrobacteria*, and *Actinobacteria* classes decreased significantly in *P. agglomerans*-treated samples, *Bacilli*, *Gammaproteobacteria*, and *Bacteroidia* had an opposite trend; notably, *Clostridia* were detected exclusively in *P. agglomerans*-treated samples, and *Blastocatellia* (Subgroup 4 and 6) in control samples. At the genus level (Figure 4c, Supplementary Table S4 and Figure S4), *Bacillus* formed the most abundant group in *P. agglomerans*-treated soil after 15 days of incubation, accounting for approximately 23.8% of the relative abundance, followed by the genus *Clostridium* (9%). In the case of the control, *Arthrobacter* and *Sphingomonas* genera were the most abundant; accounting for 23.4% and 18% of the bacterial community, respectively. Analysis at the genus level also reveals a number of other distinctions between the control and the *P. agglomerans* treated samples. For instance, the abundance of *Pontibacter, Lysobacter*, and *Domibacillus* were increased in *P. agglomerans*-treated soils compared to the control, while the relative abundance of *Microvirga, Rhizobium*, and *Rubrobacter* were decreased in *P. agglomerans*-treated soils compared to the control. The genera of *Ensifer*, *Blastococcus*, and *Sphingomonas* were only detected in control samples. These changes are also accompanied by the proliferation of a wide range of soil PSB genera in the soil of *P. agglomerans* treatment microcosms, including *Lysinibacillus* (8.74%), *Aneurinibacillus* (3.15%), *Enterobacter* (3.15%), *Acinetobacter* (4.69%), and *Enterococcus* (5.06%) (Supplementary Table S4). It is also notable that the relative abundance of *P. agglomerans* decreased by 89.01% after 15 days of incubation.

**Figure 5:**
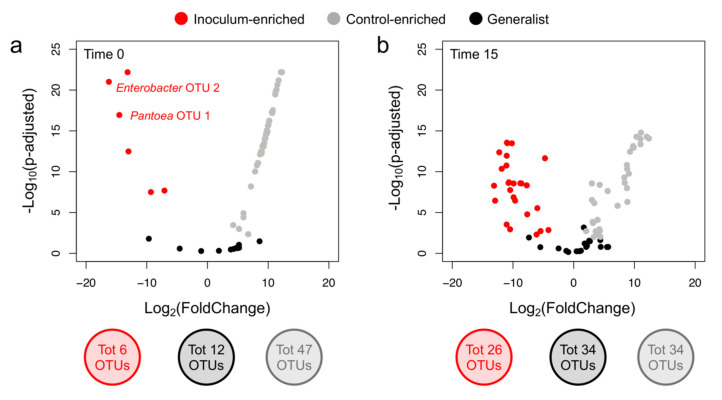
(**a**,**b**) Volcano plots showing the OTUs with differential abundance across treatment (control and inoculated) at 0 and 15 days, respectively. Number of OTUs showing similar relative abundance across treatment (generalist) and that are enriched in control or inoculated soils (control-enriched and inoculum-enriched, respectively) are reported; number of OTUs within these three categories is also reported.

We also quantified the number of OTUs that were enriched in the treatment and control soils at the two incubation times (*p*-adjusted < 0.01 and 2-fold changes in relative abundance; Figure 5). Only a small number of OTUs differentially accumulated in the inoculated soil (*n* = 6) when time 0 was considered; on the contrary, control confirmed the presence of a more diverse bacterial community with 47 enriched OTUs (Figure 5a). At the end of incubation (15 days), the number of enriched OTUs also increased in the inoculated soils (*n* = 26), confirming the bacterial community diversification mediated by *P. agglomerans* inoculation (Figure 5b). It is important to note that such changes in relative abundance could be the result of decreases/increases in several community members rather than changes in absolute abundance of specific bacterial OTUs.

**Figure 6:**
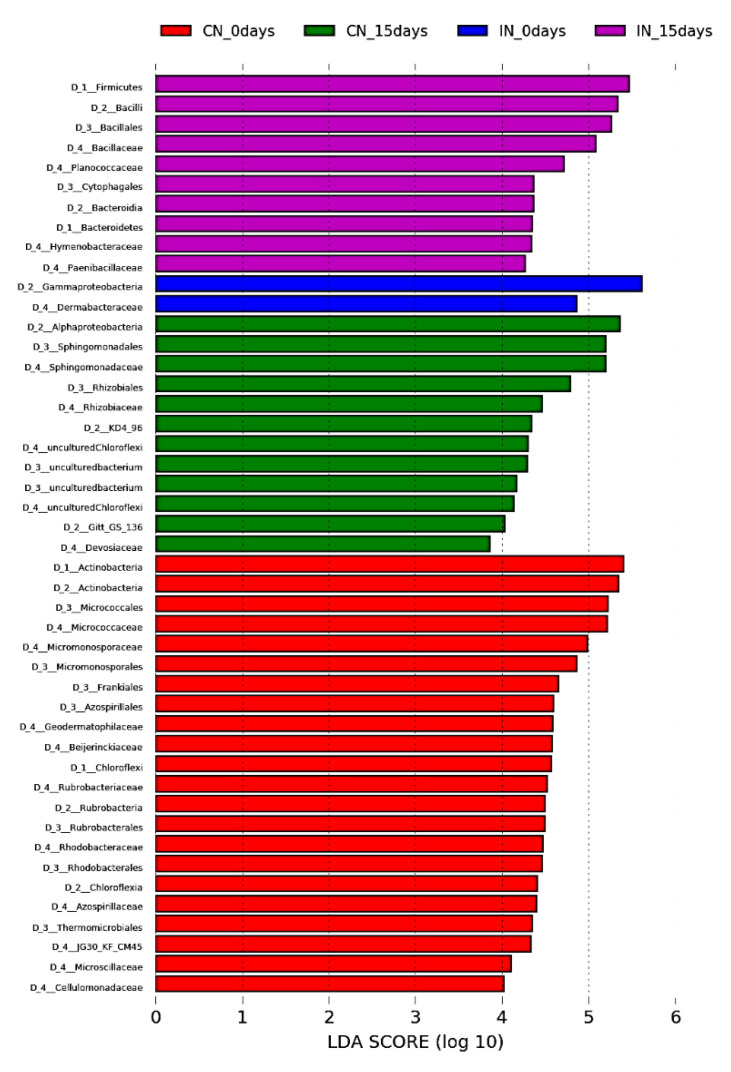
Linear discriminant analysis (LDA) scores of differentially abundant taxa in *P. agglomerans* inoculated and control sample. The LDA score indicates the effect size and ranking of each differentially abundant taxon.

Finally, we determined the bacterial discriminants for the *P. agglomerans*-treated and control soils by using LEfSe analysis; at 15 days, a total of 24 and 37 bacterial discriminants were detected in *P. agglomerans*-treated and control soils, respectively (Figure 6): members of *Bacilli* (including *Bacillus*, *Aneurinibacillus*, *Lysinibacillus*, and *Enterococcus* genera), *Clostridia* (*Clostridium* genus), *Bacteroidia* (*Pontibacter* genus), and genus DSSF69 within the *Sphingomonadaceae* family were the bacterial discriminants of PSB treatment, while Actinobacteria (including *Arthrobacter, Blastococcus, Verrucosispora,* and *Rubrobacter* genera), Acidobacteria, and Chloroflexi were enriched in the control soil group.

### 3.4. Relationships between the Soil Bacterial Community and the Critical Soil Factors

Analysis of DistLM indicated that the two abiotic variables measured (available P and pH) were significantly (*p* = 0.011 and *p* = 0.008, respectively) associated with the variation in bacterial community composition at the OTU level. The marginal and sequential stepwise tests in the DistLM analysis report the proportion of the variation explained by each variable (Table 2). The dbRDA plot showed that the first two axes of dbRDA components explain 36.1% of the total variation in the composition of the bacterial community by soil properties (Supplementary Figure S5). The first axis separated soil with *P. agglomerans* treatment at 15 days from the other soil samples. This indicated that the alteration of soil properties induced by the *P. agglomerans* application drove the soil bacterial community structure.

Pearson’s correlation coefficient analysis (Table 3) was further used to evaluate the relationships between abundant phyla (relative abundance > 1%) and soil environmental factors. It was found that the relative abundance of Firmicutes and Bacteriodetes was significantly and positively correlated with available P (*r* = 0.906, *p* = 0.001, and *r* = 0.799, *p* = 0.002, respectively) and negatively correlated with pH (*r* = −0.91, *p* = 0.001, and *r* = −0.653, *p* = 0.021, respectively). In contrast, the relative abundance of Acidobacteria, Actinobacteria, and Chloroflexi showed a significantly negative relationship with available P (*r* = −0.606, *p* = 0.037; *r* = −0.594, *p* = 0.041; *r* = −0.599, *p* = 0.039, respectively).

## 4. Discussion

Among a collection of plant growth-promoting rhizobacteria isolated from the rhizospheric soil of date palm (*Phoenix dactylifera* L.) located in the south of Tunisia, a strain identified as *P. agglomerans* V8R67 was able to develop clear phosphate solubilization zones greater than 10 mm on NBRIP agar containing Ca_3_(PO_4_)_2_. This result was in accordance with previous studies showing that members of the *Pantoea* genus display high MPS activities [15,20,29,51]. In the present work, we aimed to study the effects of the external application of *P. agglomerans* on the availability of phosphate and bacterial community of soil using high-throughput sequencing technologies. Since it has been proven that more than 99% of bacteria in a variety of environmental samples have been shown to be unculturable [52], we have adopted a culture-independent method, in which the total bacterial DNA was extracted from *P. agglomerans*-treated and control samples and the amplified bacterial 16S rRNA genes were sequenced.

We found that the relative abundance of the genus *Pantoea* significantly decreased after 15 days despite its external addition to soil samples. In general, our results are in agreement with observations from the literature that demonstrate log order scale decreases in the population size of introduced bacteria after inoculation [53]. This can be attributed to the starvation of laboratory-grown cells when introduced into the soil, and to predation and competition with the indigenous microflora [54].

Although its lower relative abundance at the end of the incubation period, results from this study demonstrated that *P. agglomerans* inoculation was an effective approach to promote the content of available P in the soil. The increase of available P following the *P. agglomerans* addition may have arisen from a combination of different mechanisms. For instance, the analyses of supernatants of growth of *P. agglomerans* showed the production of gluconic acid, directly implicated in pH drop and increase of available P. The presence of organic acid in the supernatant provided the first indication that the possible mechanism of phosphate solubilization by *P. agglomerans* is through the production of organic acids in the medium. Therefore, the *gdh* gene encoding the enzyme glucose dehydrogenase responsible for gluconic acid production and the *pqqC* gene, which catalyzes the final step of the PQQ biosynthesis, were identified in *P. agglomerans* using specific designed primers. Therefore, it was hypothesized that the capacity of *P. agglomerans* in mineral phosphate-solubilizing activity is related to the production of gluconic acid. These findings are also supported by other studies which showed that P solubilization activity seems to directly correlate to gluconic acid produced in the periplasmic space of Gram-negative bacteria [55].

Our study demonstrated the effect of *P. agglomerans* inoculum addition on changing soil factors, including available P and pH, but also on inducing modification in the structure and composition of the overall edaphic bacterial community. The abundance of the genus *Bacillus*, known as the most important PSB [55], increased in *P. agglomerans*-treated soils compared to the control. In addition, the *Clostridia* class was exclusively present in *P. agglomerans*-treated soil which also contains genera known for their P solubilization activity. The current findings are similar to those reported in field experiments based on ^31^P nuclear magnetic resonance (NMR), demonstrating an increase of available P and of phosphate solubilizing microorganisms with the application of functional PSB inoculants [56].

Soil microbial diversity and richness are considered critical soil features related to the durability of soil management activity [57,58]. The latter is usually reduced by agricultural perturbations, such as chemical P fertilizers [59]. However, alpha diversity of the bacterial community in soil treated with *P. agglomerans* was relatively similar to the control after 15 days of incubation. Moreover, the assessment of beta-diversity confirmed that *P. agglomerans* treatment does significantly affect the structure and composition of edaphic bacterial communities. In particular, differential relative abundance of certain taxa was observed; Firmicutes was the most abundant taxon in *P. agglomerans* treatment, with endospore-forming bacteria of *Bacilli* and *Clostridiales* [60] as dominant subgroups. Due to their spore formation capability, many diverse species of *Bacilli* and *Clostridia* have an advantage over other groups of microbes, in that they survive in extreme habitats for years and start to grow by using organic materials as growth substrates [61,62]. These findings were consistent with the previous study conducted by Mowlick et al. [62] which showed that members of the Firmicutes (from both *Clostridia* and *Bacilli* classes) became dominant in the edaphic bacterial community during biological soil disinfestation, suggesting their adaptation and resistance to the extreme conditions. Indeed, besides their ability to produce antagonistic compounds, these bacterial groups possess the capacity to metabolize more recalcitrant substrates, such as cellulose and lignin, which may give them an advantage in growth when sources of more easily degradable nutrients are exhausted [62,63]. By considering these characteristics of these dominant classes, we can explain the reduced presence of other groups of bacteria.

The responding microbes selected under LEfSe analysis further demonstrated that the taxa with abundant advantages in the *P. agglomerans*-treated soils were mainly related to phosphate solubilization bacteria including the genus *Bacillus*, *Lysinibacillus*, *Aneurinibacillus, Enterococcus*, and *Clostridium* [55,60,64,65,66]. The widely studied *Bacillus* genus represents one of the most diverse genera in the *Bacilli* class [67]. Several *Bacillus* strains are widely used in agriculture as plant growth-promoting and disease-suppressing agents, and a number of these strains have already been commercially developed as biological fungicides, nematicides, and insecticides [55]. *Clostridium* genus also is reported as an effective PSB [64]. *Lysinibacillus* is one of the important bacterial genera which has been known to produce secondary metabolites that can promote plant growth and enhance soil fertility [65,68]. It was also reported that *Aneurinibacillus* possessed multiple plant growth-promoting traits like production of phosphate solubilization, nitrogen fixation, indole-3-acetic acid, siderophores, HCN (hydrogen cyanide) production, and antifungal activity. Thus can be used as an effective PGP inoculant to improve crop productivity [69].

The phylum of Bacteroidetes, which was also increased in *P. agglomerans*-treated samples, was previously reported to perform functions similar to those of *Clostridiales*, such as decomposing complex plant material and producing short-chain fatty acids [70]. Within the phylum Bacteroidetes, *Pontibacter*, which was also identified as indicator phylum for *P. agglomerans*-treated soils, was reported as a potential biofertilizer to enhance soil fertility and promote plant growth [71].

It is well known that environmental factors can shape the microbial community structure [72]. Among these factors, pH and P have often been reported to be important drivers of soil bacterial community composition [73,74]. In this study, results showed that the microbial population distribution was highly related to the soil properties, which were mediated by the indirect effect of *P. agglomerans* inoculation. This hypothesis was confirmed by the significant correlations between the bacterial communities and soil properties measured. Our hypothesis is also supported by the multivariate analyses. The structure of bacterial communities was closely correlated with the soil pH and the content of available P, as has also been reported in several previous studies [75,76,77]. It was found that the relative abundance of the abundant phyla Firmicutes and Bacteroidetes showed a positive or negative correlation with available P and pH, respectively.

This finding may explain the relative abundance of both genera of *Bacillus* and *Clostridium* [13], which are significantly increased after 15 days in *P. agglomerans*-treated soils. The observed negative correlation of the genus *Clostridium* with pH was in accordance with previous reports [78]. In addition, the relative abundance of Chloroflexi, Acidobacteria, and Actinobacteria was negatively correlated with soil available P. Similarly, Zhao et al. [79] reported that the relative abundance of Chloroflexi was negatively correlated with soil available P. In the current soil microcosm, the decline of soil pH might be associated with organic acids produced during *P. agglomerans* metabolism. PSB have been shown to enhance the solubilization of P compounds with limited solubility through the release of organic acids and phosphatase enzymes [80].

Phosphorus is an essential element for all forms of life and a constituent of nucleic acids [81]. Hence, the content and availability of P might influence microbial diversity by changing the presence of specific microorganisms in soil [82]. As shown in our study, the increase in P availability after the addition of *P. agglomerans* resulted in a greatly increased abundance of PSB. Furthermore, some genera were found only in *P. agglomerans*-treated samples such as *Aneurinibacillus*, *Lysinibacillus*, *Enterococcus*, *Clostridium*, and *Pontibacter*. The increase in the abundance of inorganic PSB communities has also been reported following soil inoculation with the PSB *Pseudomonas putida* [83]. Hence, the above soil factors seemed to be of major importance in influencing the composition of soil communities, indicating that the increase in the abundance of PSB genera was controlled predominantly by abiotic mechanisms related to *P. agglomerans* addition.

It is also known that PSB can convert insoluble P to forms of soluble P in the rhizosphere. The P-solubilizing activity via the microbial biochemical activity has been found to produce and release metabolites (e.g.,citrate, malate, oxalate, and gluconate) that can acidify the microbial cells and their environment, and to produce phosphatases [84,85]. It has been shown that bacterial strains can improve P uptake by increasing P influx into the root of crops in P deficiency conditions [85]. There are few studies on P chemistry and P-containing minerals in the rhizosphere of important crops like sugarcane in P deficiency conditions and on the effects of PSB thereon [86,87]. For this reason, and for future experiments, understanding the chemical behavior of P in soils under crop cultivation is essential for appropriate P management in sustainable agricultural production. In particular, future crop experiments will enable us to better understand the plant–P-solubilizing bacteria interaction under low P availability. In this way, variations in root morphological traits along with associated rhizosphere modifications and aboveground physiological parameters related to P use efficiency could be examined. The *P. agglomerans* in the P-solubilizing capacity on the rhizosphere P availability, root morphological traits will be studied and will assist us to better understand the highly intricate root-P-solubilizing bacteria interactions under low available P forms. Such experiments will also enable us to characterize the effect that P-solubilizing bacteria have on root biophysical traits.

## 5. Conclusions

We demonstrated the direct evidence of the effect of *Pantoea* strain application on the soil microbial community. Despite the decrease of *P. agglomerans* at the end of the incubation period, the results from this study clearly demonstrate that the addition of this PSB could be a potential technological option for increasing the available P. These changes are also accompanied by the emergence and the increase of a wide range of several soil PSB genera as the indirect effect of *P. agglomerans* inoculation. Since PSB are one of the crucial determinants of plant health, the changes in native PSB communities resulting from inoculant addition will be crucial in the development of effective microbial inoculants that are favorable in enhancing soil properties. Such an approach will allow the development of inoculants better adapted to local conditions in order to increase their reliability, consistency, and efficacy. The use of microbial inoculant will reduce fertilizer and pesticide application rates and promote sustainable approaches in agriculture.

Since this study is a short-term experiment without plants, long-term microcosms and field studies of the effect of *P. agglomerans* application on the composition of the bacterial community as well as on the physicochemical parameters of the soil in field should be carried out before recommending soil management with this bacterium.

## Figures and Tables

**Table 1:** Alpha-diversity indices (richness and diversity indices) of soil samples calculated from the bacterial 16S rRNA gene sequence data. OTUs were clustered at 97% similarity level. Comparison of richness (number of OTUs observed) and diversity indices (Chao 1, ACE, Simpson, and Shannon) between control and *P. agglomerans* inoculated soil samples at time 0 and 15 days. The values are the mean ± SE, *n* = 3. For each diversity index, ANOVAs followed by the Tukey HSD test was performed; different letters in the same column indicate significant differences (*p* < 0.05).

Time	Treatment	Chao1	ACE	Simpson	Shannon
0 day	NC	53 ± 0.57 ac	53 ± 0.57 ac	0.87± 0.00 ac	4.32 ± 0.07 ac
	IN	9.66 ± 0.66 ab	9.66 ± 0.66 ab	0.55 ± 0.02 ab	1.59 ± 0.06 ab
15 days	NC	55 ± 1 b	55 ± 1 b	0.90 ± 0.02 b	4.41 ± 0.24 b
	IN	56.66 ± 1.20 c	56.66 ± 1.20 c	0.89 ± 0.02 c	4.14 ± 0.16 c

**Table 2:** Result of distance-based linear model (DistLM) analyses showing the influence of environmental parameters on bacterial soil community structure based on Bray–Curtis similarity of square-root-transformed abundance.

**Marginal Tests**							
**Variable**	**SS (trace)**	**F**	**P**	**Prop.**			
AP	10037	4.6681	0.011	0.31825			
pH	10137	4.7367	0.008	0.32142			
**Sequential Tests**							
**Variable**	**R^2^**	**SS (trace)**	**F**	**P**	**Prop.**	**Cumul**	**Res.df**
AP	0.31825	10037	4.6681	0.012	0.31825	0.31825	10
pH	0.36913	1604.8	0.72588	0.435	0.32142	0.36913	9

SS: sum of squares; F: pseudo-F; P: *p* value; Prop: proportion of explanation; Cumul: cumulative proportion of explanation; Res.df: residual degree of freedom; AP: available phosphate.

**Table 3:** Pearson’s correlation coefficients between soil environmental variable and abundant phyla (relative abundance>1%); AP: available phosphate.

Phylum	pH	AP
Acidobacteria	0.545	−0.606 *
Actinobacteria	0.574	−0.594 *
Bacteroidetes	−0.653 *	0.799 **
Chloroflexi	0.552	−0.599 *
Firmicutes	−0.910 *	0.906 **
Gemmatimonadetes	−0.279	0.103
Proteobacteria	0.385	−0.356

Significant correlation: **, *p* < 0.01 and *, *p* < 0.05.

## Supplementary Materials

The following are available online at https://www.mdpi.com/article/10.3390/microorganisms9081661/s1, Figure S1: Rarefaction curve of the bacterial 16S rRNA gene dataset before the cutting at 11,910 sequences/sample, Figure S2: PCR amplification of gdh and pqqC genes from *P. agglomerans*, Figure S3: Neighbour-joining tree based on *pqqC* and *gdh* gene sequences showing the phylogenetic relationship of strain V8R67, Figure S4: Venn diagram showing the number of shared/specific OTUs among control and inoculated soils at 0 and 15 days, respectively, Figure S5: Distance-Base Redundancy Analysis (dbRDA) ordinations of the DistLM model illustrating the relationship between the soil factors analyzed and the taxonomic composition at the OTU level in control and *P. agglomerans* inoculated sample at time point (0 and 15 days), Table S1: Primers used in this study, Table S2: Physicochemical characteristics of the soil, Table S3: Relative abundances (%) of the dominant bacterial phyla and classes in the soil samples, Table S4: Relative abundances (%) of the dominant bacterial genera in the soil samples.
