# Supplementary material for: The Impact of the Inoculation of Phosphate-Solubilizing Bacteria Pantoea agglomerans on Phosphorus Availability and Bacterial Community Dynamics of a Semi-Arid Soil
Source: Microorganisms. 2021 Aug 4;9(8):1661. doi: 10.3390/microorganisms9081661 (PMC8400695; doi:10.3390/microorganisms9081661)
Supplement: Supplementary file 1 [file microorganisms-09-01661-s001.zip › microorganisms-1276885-supplementary.pdf]

Supplementary information to manuscript

The impact of the inoculation of phosphate-solubilizing bacteria *Pantoea agglomerans* on phosphorus availability and bacterial community dynamics of a semi-arid soil

Ilhem Saadouli[1], Amor Mosbah[2], Raoudha Ferjani[1], Panagiota Stathopoulou[3], Ioannis Galiatsatos[3], Elias Asimakis[3], Ramona Marasco[4], Daniele Daffonchio[4], George Tsiamis[3,*] and Hadda-Imene Ouzari[1,*]

[1]Laboratoire Microorganismes et Biomolécules Actives (LR03ES03), Faculté des Sciences de Tunis, Université Tunis El Manar, Tunis, Tunisia

[2]Higher Institute for Biotechnology (ISBST), LR Biotechnology and Bio-Geo Resources Valorization, University of Manouba, BVBGR-LR11ES31, Biotechpole Sidi Thabet, 2020, Ariana, Tunisia

[3]Laboratory of Systems Microbiology and Applied Genomics, Department of Environmental Engineering, University of Patras

[4]Biological and Environmental Sciences and Engineering Division (BESE), Red Sea Research Center (RSRC), King Abdullah University of Science and Technology (KAUST), Thuwal, Saudi Arabia

***Correspondence:**

OUZARI Hadda-Imene: ouzari.imene@gmail.com

TSIAMIS George: gtsiamis@upatras.gr

Supplementary Tables

Supplementary Table S1. Primers used in this study

Assay	Target gene(s)	Primer name	Primer sequence (5`—3`)
PCR	*gdh*	gdh-pqqF	CTGCCAGTDAACGAYGGYCGYCTG
		gdh-pqqR	TTCRTARGTCATYGGRGTHGCCTG
PCR	*pqq*C	pqqC-OlMBF	CCCGCGAGCAGATCCAGGGCTGGGT
		pqqC-OlMBR	TAGGCCATGCTCATGGCGTC
qRT-PCR	*gdh*	gdh-F	CAGGTACTGAAACCGGCATT
		gdh-R	TACGCTGTTTCCACACCAC
qRT-PCR	*pqqC*	pqqc-F	GCTCGGAGATCAGTTCTTCA
		pqqc-R	GATTCGCTGCACCCATTTAC

Supplementary Table S2. Physicochemical characteristics of the soil.

Soil property (Unit)	Value
d (g/cm3)	0.70
dw (%)	99.51
TOM (%)	0.23
Ash (%)	99.77
C (%)	0.13
pH	7.43 ± 0.01
EC (μS/cm)	301 ± 1
CHDs (mg/kg dw)	0.41 ± 0.04
Anthrone C (mg/kg dw)	0.31 ± 0.01
Protein (mg/kg dw)	0.52 ± 0.02
TP (mg/kg dw)	0.86 ± 0.04
TKN (mg/kg dw)	10.5 ± 0.76
NH_4^+(mg/kg dw)	3.49 ± 0.22
NO_3^-(mg/kg dw)	0.07 ± 0.01

Values reported as Mean ± SE. Nitrites and water extractable phenolics were not detected. d: density; dw: dry weight; TOM Total organic matter; CHDs: total soluble carbohydrates; Anthrone C: Anthrone-reactive carbon; Protein: Bradford-reactive protein; TP: total phosphorus; TKN: Total Kjeldahl nitrogen.

Supplementary Table S3. Relative abundances (%) of the dominant bacterial phyla and classes in the soil samples (means ± SE, n = 3). Significant differences are indicated by different letters and were calculated using ANOVAs followed by the Tukey HSD test, (p-value < 0.05).

Phylum	T=0 day		T=15 day	
	CL_0	IN_0	CL_15	IN_15
Gemmatimonadetes	0.98 ± 0.11	0	1.03 ± 0.08	1.06 ± 0.46
Bacteroidetes	2.05 ± 0.36	0.008± 0.01 a	1.64 ± 0.47	5.19± 2.13a
Acidobacteria	5.39 ± 0.25a	0	8.91± 0.41a	0
Chloroflexi	7.22 ± 0.69	0	6.73 ± 0.90a	0.45 ± 0.11a
Actinobacteria	50.12 ± 1.26a	0.77 ± 0.33	32.26± 5.11ab	1.65± 1.30b
Firmicutes	2.9± 0.40 c	17.1 ± 1.67 b	2.9 ± 0.08 a	61.99 ± 12.02abc
Proteobacteria	31.31 ± 0.43	82.1 ± 1.71 a	46.49 ± 3.31	29.63 ± 13.13a

Class	T=0 Day		T=15 Days	
	CL_0	IN_0	CL_15	IN_15
Anaerolineae	0.04±0.00	0	0.033±0.02	0.037±0.02
Gitt.GS.136	0.62±0.08b	0	0.85±0.10 a	0.06±0.04 ab
Blastocatellia	0.70±0.05a	0	1.42±0.03 a	0
Gemmatimonadetes	0.98±0.11	0	1.034±0.08	1.06±0.46
KD4.96	1.68±0.22b	0	2.16±0.11 a	0.08±0.02 ab
Chloroflexia	4.87±0.40b	0	3.68±0.96 a	0.26±0.06 ab
Bacteroidia	2.05±0.36	0.008±0.01a	1.64±0.47	5.19±2.13 a
Rubrobacteria	5.65±0.27bc	0	3.31±0.40 ab	0.14±0.04 ac
Subgroup.6	4.68±0.21a	0	7.48±0.39 a	0
Clostridia	0	0	0	16.3±0.70
Bacilli	2.90±0.40b	17.10±1.67 c	2.90±0.08 a	45.69±12.37 abc
Actinobacteria	44.47±1.39acd	0.77±0.33 ab	28.95±4.98 bde	1.51±1.35 ce
Alphaproteobacteria	31.23±0.44bce	0.006±0.01 de	44.77±1.67 abd	9.15±3.99 ac
Gammaproteobacteria	0.08±0.02 c	82.09±1.71 abc	1.71±1.67 b	20.47±14.26 a

Supplementary Table S4. Relative abundances (%) of the dominant bacteria genus in the soil samples (means ± SE, n = 3) Significant differences are indicated by different letters ANOVAs followed by the Tukey HSD test, (p-value < 0.05).

Genera	T=0 Day		T=15 Days	
	CL_0	IN_0	CL_15	IN_15
Geodermatophilus	1.67±0.02 bc	0	0.81±0.13 ab	0.03±0.01 ac
Domibacillus	0.55±0.05	0	0.57±0.016	1.51±1.39
Unc.*Acidobacteria*	1.18±0.02 a	0	1.47±0.37 a	0
Ensifer	0.92±0.03 a	0	2.15±0.35 a	0
Aneurinibacillus	0	0	0	3.15±0.84
Lysobacter	0	0	0.10±0.10	3.17±2.11
Unc.*Chloroflexi*	1.94±0.21 b	0	1.37±0.07 a	0.10±0.03 ab
Solibacillus	0	1.47±1.22	0	1.95±1.86
Unc. BacteriaChloroflexi	1.68±0.21 b	0	2.16±0.11 a	0.08±0.02 ab
Allorhizobium.	0.04±0.02	0	3.98±3.89	0.196±0.11
Acinetobacter	0	0	0	4.69±4.69
Pontibacter	0.03±0.01	0	0.13±0.06	5.19±2.13
Unc.BacteriaAcidobacteria.	2.07±0.2 a	0	3.46±0.12 a	0
Paracoccus	3.91±0.31 ab	0	1.22±0.52 a	0.46b±0.41
DSSF69	0	0	0	5.69±2.95
Lysinibacillus	0	0	0	8.74±4.81
Blastococcus	5.77±0.26 a	0	3.19±0.51 a	0
Rubrobacter	5.65±0.26 bc	0	3.31±0.39 ab	0.14±0.04 ac
Clostridium	0	0	0	9.49±0.71
Planomicrobium	0	9.99±2.45	0	0
Enterococcus	0	5.42±1.45	0	5.06±2.98
Microvirga	7.39±0.17 bc	0	4.56±0.79 ab	0.46±0.14 ac
Uncultured	7.30±0.71 b	0	12.5±2.24 a	0.27±0.14 ab
Enterobacter	0	19.49±1.75	0	3.15±3.15
Sphingomonas	8.41±0.17 a	0	18.04±2.65 a	0
Bacillus	2.28±0.41	0.17±0.14	2.08±0.15	23.82±16.63
Arthrobacter	34±1.12	0.35±0.32cd	23.49±4.50 ac	1.38±1.38 ab
Pantoea	0	62.44±2.69 a	0	6.86±6.86 a

Supplementary Figures

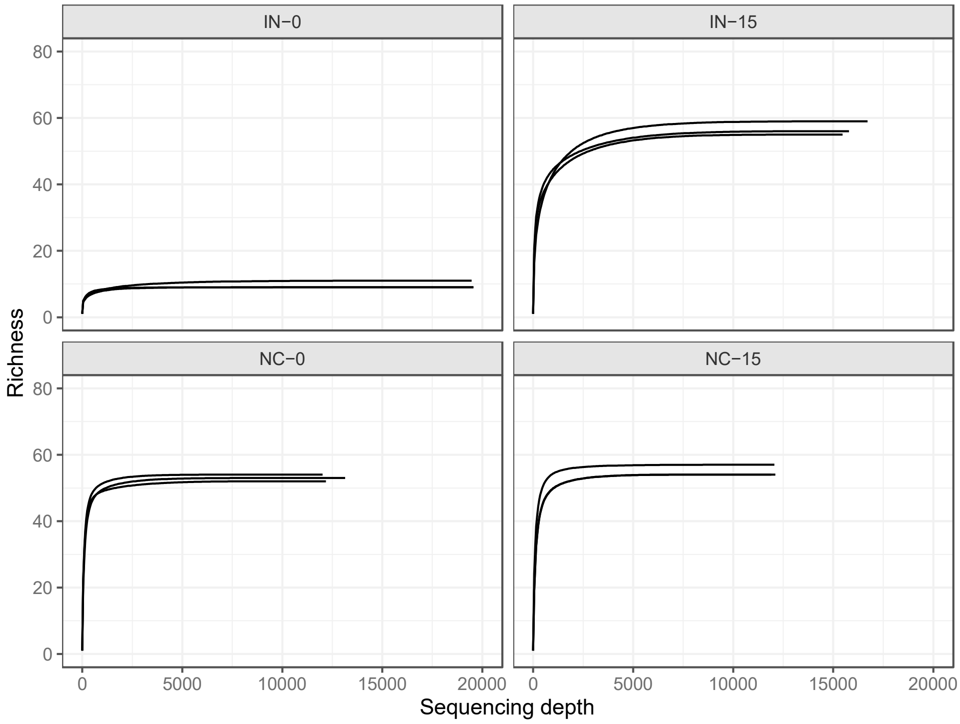

Supplementary Figure S1. Rarefaction curve of the bacterial 16S rRNA gene dataset before the cutting at 11,910 sequences/sample.

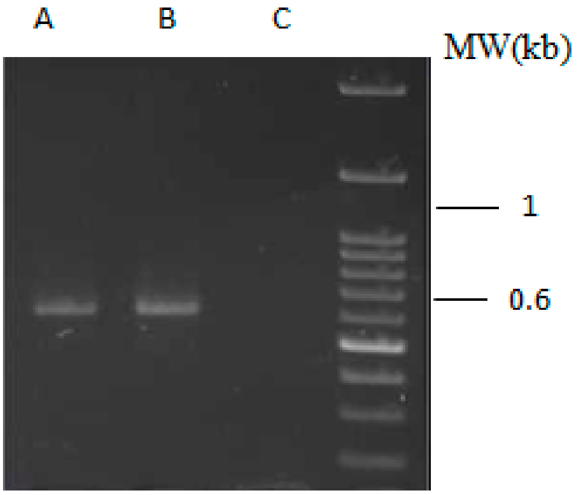

Supplementary Figure S2. PCR amplification of *gdh* and *pqqC* genes from *P. agglomerans*. Agarose gel electrophoresis showing amplification of *gdh* (A) and *pqqC* (B) genes from *P. agglomerans* (C) Negative control. Lane MW: 1 kb DNA Ladder.

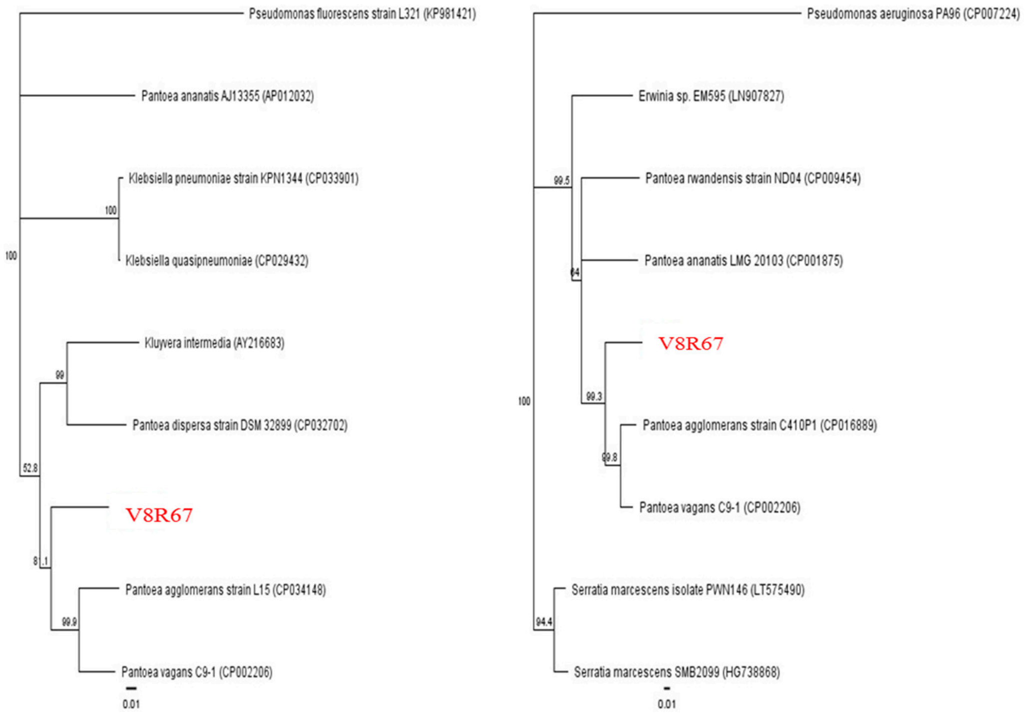

Supplementary Figure S3. Neighbour-joining tree based on *pqqC* (left tree) and *gdh* (right tree) gene sequences showing the phylogenetic relationship of strain V8R67. *Pseudomonas* species was used as an outgroup. The numbers at the nodes indicate the levels of bootstrap support based on data for 1000 replicates.

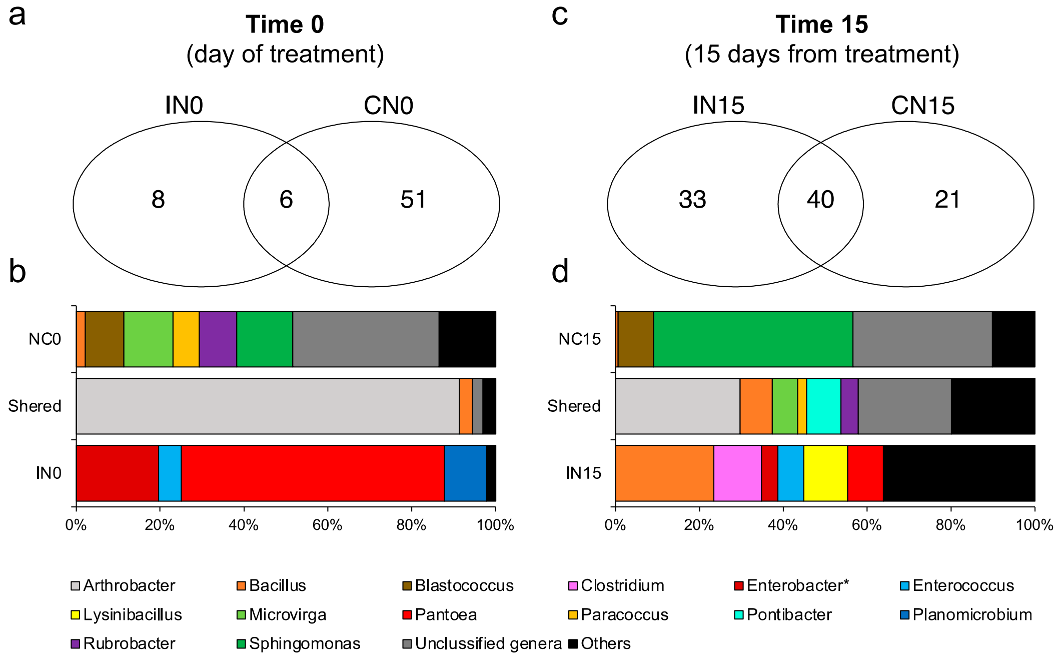

Supplementary Figure S4. (**a** and **c**) Venn diagram showing the number of shared/specific OTUs among control and inoculated soils at 0 and 15 days, respectively. (**b** and **d**) Relative abundance of shared, control-specific, and inoculated-specific bacterial taxa (genus level) at

time 0 and 15, respectively. OTUs that did not show classification at the genus level and had low abundance (<1%) are grouped as 'Unclassified genera' and 'Others', respectively.

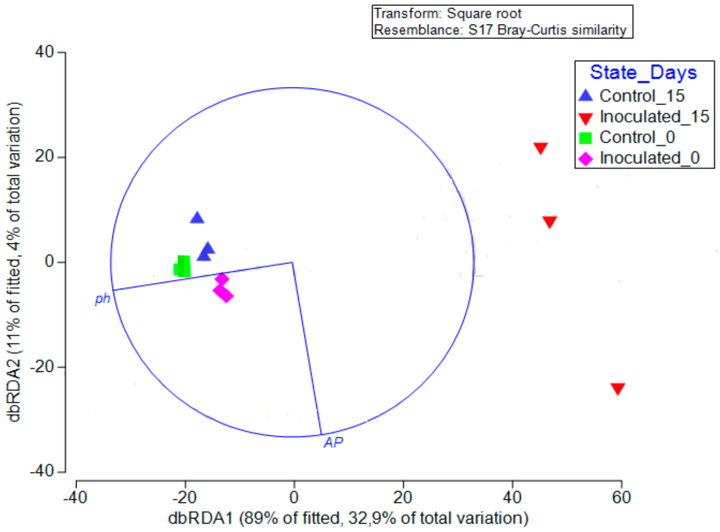

Supplementary Figure S5. Distance-Base Redundancy Analysis (dbRDA) ordinations of the DistLM model illustrating the relationship between the soil factors analyzed and the taxonomic composition at the OTU level in control and *P. agglomerans* inoculated sample at time point (0 and 15 days). Vector overlays show the strength of the relationship between the variables and the dbRDA axes. Axis legends include percentage of variation explained by the fitted model and percentage of total variation explained by the axis.
